# Axillary Recurrence and Survival After Omission of Axillary Lymph Node Dissection in Patients with Breast Cancer and Micrometastases or Isolated Tumor Cells in the Sentinel Node: A Danish National Register Study

**DOI:** 10.1245/s10434-026-20072-x

**Published:** 2026-07-06

**Authors:** Riazan Heidari Rosenlund Korsholm, Niels Kroman, Tove F. Tvedskov, Frederikke Munck

**Affiliations:** 1 Department of Breast Surgery, Copenhagen University Hospital–Gentofte, Hellerup, Denmark; 2 Department of Surgery, Zealand University Hospital, Køge, Denmark; 3 Faculty of Health and Medical Sciences, University of Copenhagen, Copenhagen, Denmark

**Keywords:** Breast cancer, Breast surgery, Sentinel lymph node biopsy, Axillary lymph node dissection, Micrometastases, Isolated tumor cells, Axillary recurrence, Survival

## Abstract

**Background:**

Completion axillary lymph node dissection (ALND) is no longer routinely recommended for patients with breast cancer and minimal nodal involvement. In 2012, Danish national guidelines were revised to omit ALND for patients with sentinel node (SN) micrometastases (pN1mi) or isolated tumor cells (pN0[i+]). Long-term evidence on axillary recurrence (AR) and overall survival (OS), particularly after mastectomy, remain limited. This nationwide retrospective register-based study evaluated long-term outcomes after omission of ALND.

**Methods:**

Clinically node-negative women with pN1mi/pN0(i+) diagnosed between 2008 and 2021 were identified in the Danish Breast Cancer Group database. Patients treated before 2012 (ALND group) were compared with those treated after guideline revision in 2012 (no-ALND group). Both groups were compared with node-negative patients (pN0) treated after 2012. The primary endpoint was AR, and the secondary endpoint was OS. Survival was analyzed using Kaplan–Meier and multivariable Cox regression.

**Results:**

Among 22,253 eligible patients, 5066 had pN1mi/pN0(i+). The median follow-up period was 6.4 years. Axillary recurrence occurred for 1.1% of the patients overall and was more frequent among the patients without ALND than among those with ALND (1.5% vs 0.2%; *p* < 0.001). Among the pN0 patients without ALND, 0.5% experienced AR. Long-term OS for the pN1mi/pN0(i+) patients was 81.3% (95% confidence interval, 79.9–82.7), with no significant difference after adjustment for prognostic factors (*p* = 0.6). Mastectomy-specific analysis showed no difference in OS (*p* = 0.08).

**Conclusion:**

Omission of ALND for patients with minimal SN involvement was associated with a slightly higher rate of AR but no adverse impact on long-term OS. These findings support continued de-escalation of axillary surgery, even for selected patients undergoing mastectomy.

Axillary staging is central to the management of patients with breast cancer, providing prognostic information used in adjuvant treatment decisions.

With the introduction of the sentinel lymph node biopsy (SLNB) in the late 20th century, pathologists gained the ability to examine lymph nodes in greater detail, leading to detection of a greater number of small metastases. Approximately 15% of patients have only micrometastases (pN1mi) or isolated tumor cells (pN0[i+]) in the sentinel node [SN]).^1^ Among patients with this minimal metastatic burden, only 10–20% have metastases in non-sentinel nodes, rendering completion axillary lymph node dissection (ALND) unnecessary in most cases.^2,3^ Consequently, SLNB has replaced completion ALND for axillary staging in clinically node-negative patients with minimal nodal disease, such as pN1mi or pN0(i+).

In 2004,^4^ SLNB was fully implemented across Danish breast cancer centers following the national guidelines for breast cancer management, which in Denmark are defined by the Danish Breast Cancer Group (DBCG).^5^ Each year, approximately 3700 Danish patients undergo SLNB.^6^ In 2012, the DBCG guidelines were revised to omit ALND for patients with pN1mi or pN0(i+) in the SN at primary surgery.^2,7^ This paradigm shift from SLNB with ALND to SLNB alone has spared an estimated 550 Danish women annually from ALND and its associated upper-limb morbidities, including pain, paresthesia, lymphedema, and functional impairment.^3,8–10^

The de-escalation of axillary surgery for patients with minimal metastatic disease has been supported by several trials, including IBCSG 23-01,^11,12^ AATRM,^13^ and a nationwide Danish study.^2^ These studies found no deterioration of prognosis when ALND was omitted for patients with minimal axillary metastatic burden (pN1mi or pN0[i+]) in the SN.^3^ Even with 10–20% having residual axillary disease, these patients did not tend to experience axillary recurrence (AR) due to sufficient adjuvant treatment after primary surgery.

However, an important limitation of previous trials has been the underrepresentation of patients undergoing mastectomy. This subgroup is of particular interest because they generally do not receive adjuvant radiotherapy, which otherwise contributes to axillary control after breast-conserving surgery. Thus, the safety of omitting ALND for patients undergoing mastectomy remains less well-established.^14^

Furthermore, questions remain regarding whether follow-up evaluation has been sufficient to detect late AR.^10,15,16^ Andersen et al.^17^ emphasized the importance of long-term follow-up evaluation for patients with limited axillary disease because smaller metastases may manifest only after prolonged periods.

Although several prospective and randomized studies have demonstrated the safety of omitting ALND for patients with minimal sentinel node involvement, long-term real-world data remain limited, particularly for patients undergoing mastectomy and for populations treated after implementation of guideline-based omission of ALND in routine clinical practice.

To assess the long-term safety of omitting ALND for patients with minimal metastatic disease in the SN at up-front surgery, including those undergoing mastectomy, we investigated AR and overall survival (OS) in a large national cohort of Danish patients with breast cancer and pN1mi or pN0(i+) with and without ALND after long-term follow-up evaluation using data from the DBCG database.

## Materials and Methods

### Study Design and Population

This nationwide, retrospective, register-based study included all women with clinically node-negative primary breast cancer and pN1mi or pN0(i+) in the SN, as well as a group of node-negative (pN0) patients who underwent surgery after 2012 used for comparison. All the patients were treated between 1 January 2008, and 31 December 2021, at 1 of 16 Danish surgical departments and underwent either breast-conserving surgery (BCS) or mastectomy.

The patients treated before 2012 (ALND group) were compared with those treated after 2012 (no-ALND group) following implementation of the new DBCG guideline. The patients with pN0 disease treated after 2012 without ALND served as a reference group.

The exclusion criteria ruled out macrometastases in the SN, receipt of neoadjuvant therapy, omission of ALND before 2012, and performance of ALND after 2012. Patients undergoing any surgical procedure other than mastectomy or BCS were excluded.

### Data Acquisition

In Denmark, histopathologic and clinical data, treatment, and follow-up evaluation have been prospectively registered in a national database managed by the DBCG since its foundation in 1977.^5^ Data for this study were obtained from the DBCG database, and the variables extracted included patient and tumor characteristics such as age at diagnosis, type of primary surgery, date of surgery, ALND (yes/no), number of SNs with/without macrometastases, pN1mi or pN0(i+), tumor size, histologic type, malignancy grade, estrogen receptor (ER) status, human epidermal growth factor receptor 2 (HER2) status, adjuvant systemic treatment, radiotherapy, and information regarding recurrence, including type and date of the event.

Data were retrieved from the DBCG database on 4 July 2022. Missing data were sought in the original patient files. Definitions of pN1mi and pN0(i+) were according to the eighth edition of the American Joint Committee on Cancer (AJCC) staging manual.^18^

### Statistical Analysis

The primary endpoint was AR, and the secondary endpoint was OS. Axillary recurrence was defined as any ipsilateral axillary lymph node recurrence, regardless of the presence of recurrence at other sites.

Descriptive statistics, chi-square tests, and Fisher’s exact tests were used to compare patient and tumor characteristics. Cumulative AR was estimated, and OS was assessed with Kaplan–Meier analysis and the log-rank test. A multivariable Cox proportional hazards model evaluated the association between ALND and OS, adjusting for age, histology, grade, ER and HER2 status, adjuvant systemic therapy, and radiotherapy (Table 1).

**Table 1:** Baseline clinicopathologic characteristics according to nodal status and ALND status of 22,253 Danish patients with breast cancer

Variables	ALND-group before 2012 *n* (%)	No-ALND group after 2012 *n* (%)	Node-negative no-ALND group *n* (%)
Total	1441	3625	17187
*Age (years)*			
<40	51 (3.5)	111 (3.1)	414 (2.4)
40–49	215 (14.9)	474 (13.1)	1658 (9.6)
50–59	449 (31.2)	866 (23.9)	4202 (24.4)
60–69	559 (38.8)	1078 (29.7)	6252 (36.4)
≥70	167 (11.6)	1096 (30.2)	4661 (27.1)
*Histology*			
Ductal	1184 (82.2)	2815 (77.7)	13228 (77.0)
Lobular	168 (11.7)	534 (14.7)	1926 (11.2)
Other	89 (6.2)	276 (7.6)	2033 (11.8)
*Grade*			
1	454 (31.5)	928 (25.6)	5370 (31.2)
2	642 (44.6)	1877 (51.8)	7400 (43.1)
3	267 (18.5)	675 (18.6)	3061 (17.8)
Unknown	78 (5.4)	145 (4.0)	1356 (7.9)
*Subtype*^a^			
ER+/HER2–	1079 (74.9)	3032 (83.6)	13966 (81.3)
ER+/HER2+	121 (8.4)	307 (8.5)	1246 (7.2)
ER–/HER2+	50 (3.5)	78 (2.2)	371 (2.2)
Double-negative	90 (6.2)	178 (4.9)	1449 (8.4)
Unknown	101 (7.0)	30 (0.8)	155 (0.9)
*Tumor size (cm)*			
≤1	283 (19.6)	647 (17.8)	5940 (34.6)
>1–2	702 (48.7)	1842 (50.8)	8127 (47.3)
>2–3	337 (23.4)	777 (21.4)	2295 (13.4)
>3	116 (8.0)	356 (9.8)	813 (4.7)
Unknown	3 (0.2)	3 (0.1)	12 (0.1)
*Type of surgery*			
Mastectomy	406 (28.2)	1053 (29.0)	2941 (17.1)
BCS	1035 (71.8)	2572 (71.0)	14246 (82.9)
*Locoregional radiotherapy*			
No	32 (2.2)	174 (4.8)	1182 (6.9)
Yes	954 (66.2)	2282 (63.0)	12222 (71.1)
Unknown	455 (31.6)	1169 (32.2)	3783 (22.0)
*Chemotherapy*			
Yes	647 (44.9)	1326 (36.6)	4523 (26.3)
No	51 (3.5)	234 (6.5)	901 (5.2)
Unknown	743 (51.6)	2065 (57.0)	11763 (68.4)
*Endocrine therapy*			
Yes	1128 (78.3)	2879 (79.4)	11353 (66.1)
No	13 (0.9)	102 (2.8)	605 (3.5)
Unknown	300 (20.8)	644 (17.8)	5229 (30.4)
*HER2-targeted therapy*			
Yes	114 (7.9)	277 (7.6)	1146 (6.7)
No	6 (0.4)	36 (1.0)	217 (1.3)
Unknown	1321 (91.7)	3312 (91.4)	15824 (92.1)

ALND, Axillary lymph node dissection; ER, Estrogen receptor; HER2, Human epidermal growth factor receptor 2; BCS, Breast-conserving surgery

^a^Breast cancer subtype was defined using ER and HER2 status only. “Double-negative” therefore refers to ER-negative/HER2-negative disease because progesterone receptor (PR) status was not included in the subtype definition. HER2-targeted therapy had a high proportion of unknown values and should be interpreted as recorded treatment status rather than complete treatment ascertainment.

A two-tailed alpha of 0.05 was considered statistically significant. All analyses were performed using R statistical software (R Core Team, 2021, Vienna, Austria).^19^

### Study Approvals

The study was approved by the Center for Health, Capital Region of Denmark (j.no. R-22034923) and The Research Project Registration and Data Management Team, Capital Region of Denmark (j.no. P-2022-388). Patient consent was waived in accordance with Danish legislation on registry-based research.

## Results

The study obtained data on 36,716 patients who underwent primary surgery between 1 January 2008 and 31 December 2021 at 1 of 16 Danish departments. The study excluded ineligible patients, including 9633 patients with macrometastases, 3890 patients who had surgery after 2012 and did undergo an ALND, 403 pN0 patients before 2012, 505 patients who received treatment other than a BCS or mastectomy, and 32 patients after 2012 who had an ALND due to “other causes” such as suspicion of metastases during surgery or no SN found during surgery. After the exclusions, 22,253 patients were included in the analyses.

**Fig. 1:**
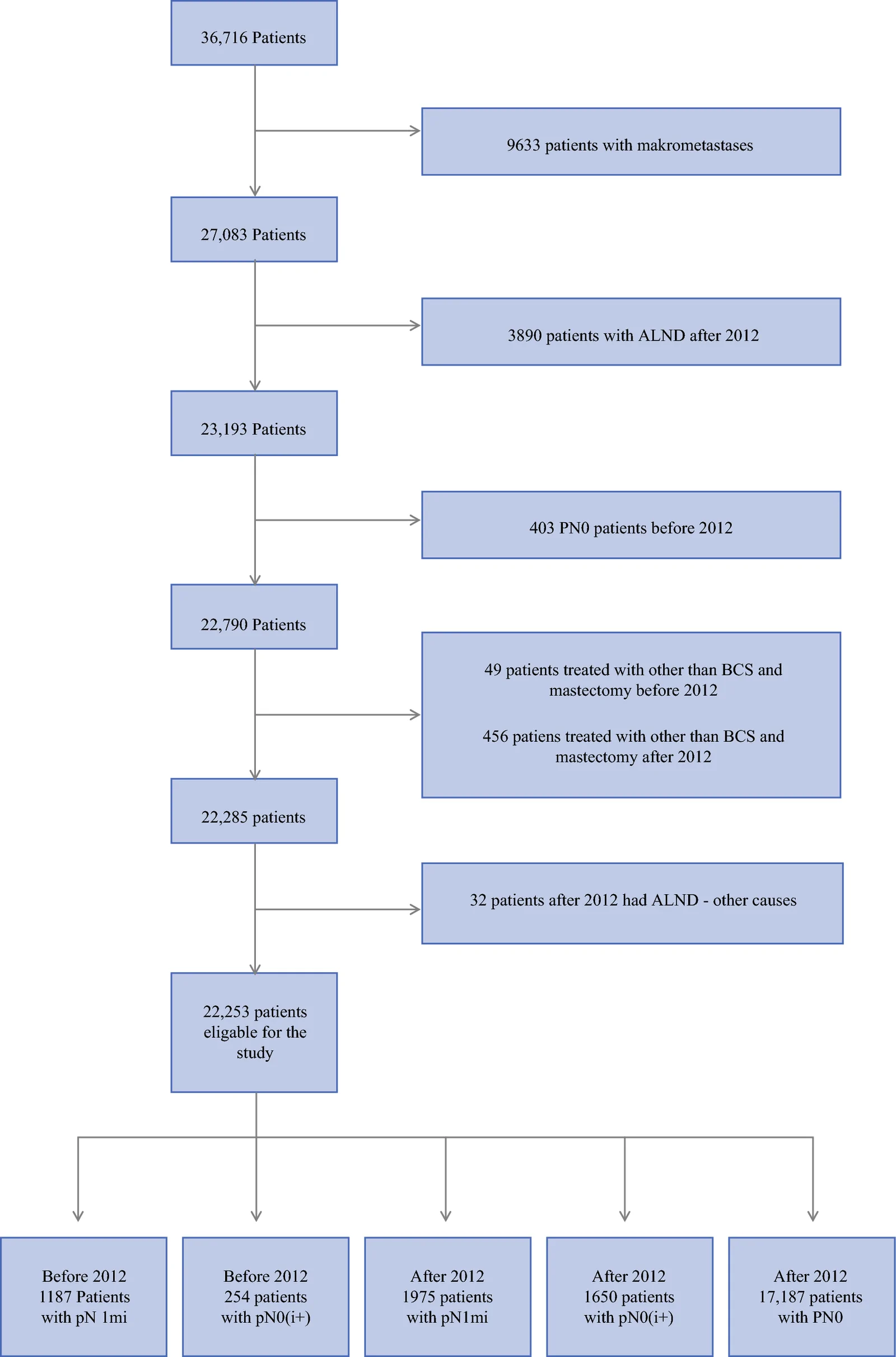
Exclusion criteria of patient enrollment

Of the group of patients who underwent surgery before 2012 and had a SLNB and an ALND, 1187 patients had pN1mi and 254 patients had pN0(i+) in the SN. In the group of patients who had surgery after 2012 and had an SLNB without an ALND, 1975 patients had pN1mi and 1650 patients had pN0(i+) in the SN. The group of pN0 patients consisted of 17,187 patients who underwent surgery after 2012 and had no metastases in the SN and therefore no ALND. See Fig. 1 for details of exclusion as well as patient and tumor characteristics.

The median follow-up period for the patients with AR was 4.2 years (95% confidence interval [CI], 3.1–5.3 years). Among the patients who experienced AR, the median time from primary surgery to AR was 2.8 years (interquartile range [IQR], 1.7–4.7 years). Axillary recurrence occurred for only 1.1% of all the patients with pN1mi or pN0(i+). Analysis of the patients with pN1mi or pN0(i+) with or without ALND showed that the patients without ALND had a significantly higher risk of AR (1.5%) than the patients with ALND (0.2%) (*p* < 0.001). In a subanalysis of patients who had pN1mi with or without ALND, a similar difference was observed (0.09% and 1.1%, respectively; *p* < 0.01). In a subanalysis of the patients with pN0(i+), none of the patients with pN0(i+) and ALND had an AR, whereas 0.9% of the patients with pN0(i+) had an AR when ALND was omitted (*p =* 0.16). This difference was not significant. For comparison, in the group of pN0 patients without ALND, 0.5% had an AR. The results of the AR analysis are shown in Table 2.

**Table 2:** Axillary recurrence (AR) in 22,253 Danish patients with breast cancer and micrometastases, isolated tumor cells, or no metastases in the sentinel node (SN)

	Micrometastases	*p* Value		Isolated tumor cells	*p* Value		Node-negative
AR	Axillary lymph node dissection						
	Yes (before 2012)	No (after 2012)		Yes (before 2012)	No (after 2012)		No (after 2012)
	0.09%	1.1%	*P* < 0.01	0%	0.9%	*p* = 0.16	0.5%

Regarding OS, the median follow-up period for the entire cohort including the pN0 patients, was 6.36 years (95% CI, 6.28–6.45 years). As expected, the median follow-up period for the patients treated before 2012 (13.07 years; 95% CI, 13.01–13.18 years) was significantly longer than for patients treated after 2012 (5.97 years; 95% CI, 5.86–6.11 years) (*p* < 0.01). The median follow-up period for the pN0 patients was 6.04 years (95% CI, 5.98**–**6.12 years).

**Fig. 2:**
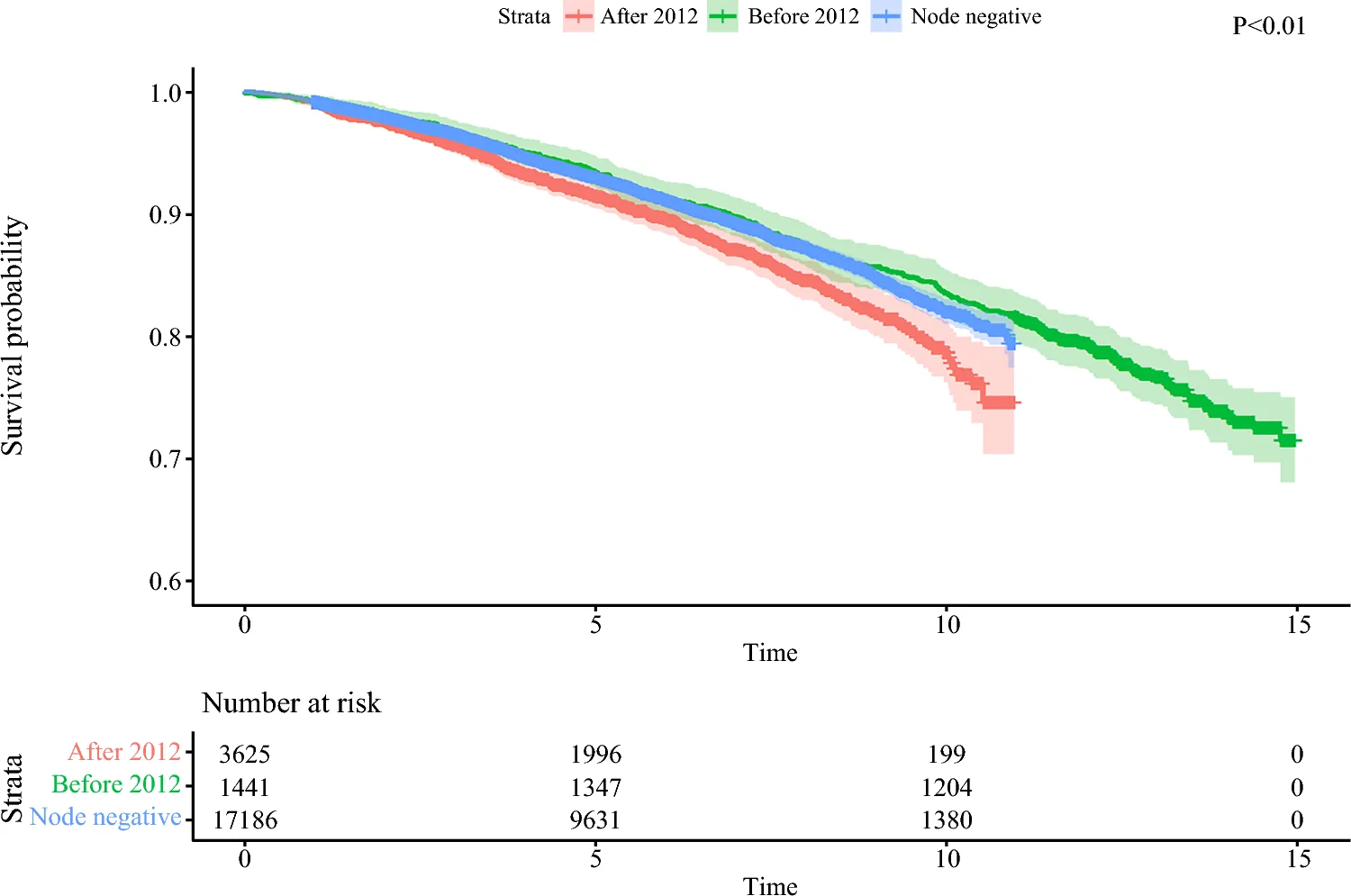
Kaplan-Meier survival curves; time in years; survival probability for 22,253 Danish patients with breast cancer who underwent surgery after 2012 (no-ALND group [*red*]) and those who had surgery before 2012 (ALND-group [*green*]); node-negative patients after 2012 (no-ALND ([*blue*]). ALND, axillary lymph node dissection

The estimated OS at 10 years for the whole cohort of patients with pN1mi or pN0(i+) was 81.3% (95% CI, 79.9–82.7%). In the unadjusted analysis, OS was significantly lower for the patients without ALND (78.8%; 95% CI, 76.6–81.3%) than for the patients with ALND (83.6%; 95% CI, 81.7–85.5%) (*p* < 0.001). For comparison, the OS for the patients with pN0 was 82% (95% CI, 81.0–83.1%). Kaplan-Meier survival curves are shown in Fig. 2, and the unadjusted OS is shown in Table 3.

**Table 3:** Unadjusted 10-year overall survival of 22,253 Danish patients with breast cancer and micrometastases, isolated tumor cells, or no metastases in the sentinel node (SN)

	Patients with micrometastases and isolated tumor cells With ALND (95% CI)	Patients with micrometastases and isolated tumor cells Without ALND (95% CI)	Patients with node- negative disease Without ALND (95% CI)	*p* Value
Estimated overall survival at 10 years (unadjusted)	0.84 (0.82–0.86)	0.79 (0.76–0.81)	0.82 (0.81–0.83)	*p* < 0.001

ALND, Axillary lymph node dissection; CI, Confidence interval

When the analysis adjusted for risk factors such as age at diagnosis, histologic type, malignancy grade, ER status, HER2 status, tumor size, adjuvant regimen, and radiation, no statistically significant difference in OS was observed between the patients with pN1mi or pN0(i+) who had ALND and those who did not (*p =* 0.6). Likewise, no statistically significant difference in OS was found for subgroups of pN1mi with and without ALND (*p =* 0.5) or for subgroups of pN0(i+) with and without ALND (*p =* 0.6). The results of adjusted Cox proportional hazard ratios for death are shown in Table 4.

**Table 4:** Adjusted hazard ratios for death of 5066 Danish patients with breast cancer and micrometastases and/or isolated tumor cells in the sentinel node^a^

	Patients with micrometastases and isolated tumor cells HR (95% CI	Patients with micrometastases HR (95% CI)	Patients with isolated tumor cells HR (95% CI)
Adjusted HR for death (no-ALND vs. ALND)	0.95 (0.79–1.15) *p =* 0.58	0.92 (0.75–1.14) *p =* 0.46	0.89 (0.60–1.32) *p =* 0.56

HR, Hazard ratio; CI, Confidence interval; ALND, Axillary lymph node dissection; ER, Estrogen receptor; HER2, Human epidermal growth factor receptor 2

^a^Patients who underwent surgery before 2012 (ALND group) were compared with patients who underwent surgery after 2012 (no-ALND group). Patients who underwent surgery before 2012 (ALND group) were used as the reference group. Adjustments were made for: age, histology, tumor grade, ER status, HER2 status, medical treatment, loco-regional radiotherapy, and tumor size.

Subsequently, a subanalysis was performed for the patients who underwent mastectomy as their primary surgery. Of the 1459 patients (29%) who underwent mastectomy, 406 had surgery before 2012, comprising 331 patients with pN1mi and 75 patients with pN0(i+). A total of 1053 patients underwent a mastectomy after 2012, comprising 541 patients with pN1mi and 512 patients with pN0(i+).

The median follow-up period for all the patients who underwent mastectomy was 7.58 years (95% CI, 7.18–7.97 years). The patients treated after 2012, had a shorter median follow-up period of 5.75 years (95% CI, 5.44–6.04 years) compared with 13.24 years (95% CI, 13.07–13.43 years) for those treated before 2012.

No patients in the mastectomy group with pN0(i+) had an AR. Only 1 patient (0.3%) with pN1mi who underwent surgery before 2012 had an AR, whereas 17 patients (3.1%) with pN1mi who underwent surgery after 2012, had an AR. Analysis of the patients with pN1mi showed a statistically significant difference between the patients who had surgery before 2012 and those who had surgery after 2012 (*p* < 0.01).

At 10 years, OS for the subgroup of patients who underwent mastectomy after 2012 was 70.4% (95% CI, 65.9–75.2%), whereas it was 75.4% (95% CI, 71.3–79.7%) for those treated before 2012. No statistically significant difference in OS was observed between the two groups (*p =* 0.08).

## Discussion

This nationwide, register-based study of 5066 Danish women with minimal axillary nodal involvement (pN1mi or pN0[i+]) at primary surgery for breast cancer found that omission of ALND did not adversely affect long-term outcomes. Although a slightly higher AR rate was observed after the guideline change in 2012, with omission of ALND, the absolute number of events remained low and comparable with that seen for the pN0 patients. Importantly, this difference did not translate into reduced OS. The observed AR rate after omission of ALND (1.5%) aligns with prior reports of 1% to 2% in similar populations and supports the continuing shift toward less invasive axillary management.^2,3,5,6,13–15,17^

Our findings are consistent with those of previous randomized and prospective studies supporting omission of completion ALND for patients with minimal metastatic burden, especially pN1mi. The IBCSG 23-01 trial of 934 patients with 9.7 years of follow-up evaluation demonstrated non-inferior disease-free survival (DFS) and OS without ALND, reporting an AR of 1.7% and markedly reduced morbidity.^11^

Similarly, the Spanish AATRM trial, which included 233 patients with breast cancer and pN1mi in their SN, found after a median follow-up period of 62 months that 2.5% of the patients without an ALND had an AR, compared with 1% of the patients who had undergone an ALND. They found no significant difference in DFS between the two groups.^13^

Early results from the Swedish SENOMIC prospective multicenter trial included 566 patients with BCS or mastectomy with pN1mi. The 3 year follow-up evaluation showed an excellent event-free survival rate, but the analysis found a higher recurrence rate among mastectomy patients who did not receive adjuvant radiotherapy.^16^

In the latest extended SENOMIC analysis, investigators focused exclusively on mastectomy patients (*n* = 455) who did not undergo completion ALND, most of whom did not receive nodal radiotherapy. After a median follow-up period of 53 months, the 5 year event-free and cancer-specific survival rates were 86.8% and 97.0%, respectively. Although the isolated AR rate (3.1%) was slightly higher than in earlier trials, this did not translate into worse survival. This updated analysis provides the strongest prospective evidence to date supporting omission of ALND for patients treated with mastectomy, a subgroup comparable with ours.^14^

The increased risk of AR observed after omission of ALND, particularly among patients undergoing mastectomy, should be interpreted with caution. Although the absolute recurrence rate remained low, AR may require additional surgery, radiotherapy, and systemic treatment, potentially increasing morbidity. Nevertheless, the low event rate and absence of an adverse impact on OS survival suggest that omission of ALND remains appropriate for carefully selected patients with limited nodal disease.

In Denmark, omission of ALND for patients with pN1mi or pN0(i+) in the SN has previously been investigated by Tvedskov et al.^2^ Their study included 2074 patients with breast cancer and pN1mi or pN0(i+) treated before the change in guidelines in 2012, with ALND omitted for selected patients. They found comparable OS between groups with and without ALND, and an AR rate of only 1.6% after a median follow-up period of 6 years and 3 months.

The current study confirmed these previous Danish results in a larger post-implementation cohort and with a long-term median follow-up period for the patients before (13.07 years) and after (5.97 years) 2012. It also complemented the existing prospective evidence by providing long-term real-world outcome data from a large national cohort treated in routine clinical practice, including a substantial subgroup of patients undergoing mastectomy. In total, these results validate the safety of omitting ALND in up-front surgery for patients with minimal nodal involvement, including those undergoing mastectomy, and align with the growing international evidence supporting de-escalation of axillary surgery.

A major strength of this study was its nationwide design and nearly complete follow-up evaluation, minimizing selection bias and ensuring high external validity. The comprehensive DBCG database allowed detailed adjustment for patient, tumor, and treatment variables. In addition, the large cohort and inclusion of a substantial subgroup of patients undergoing mastectomy provided important real-world long-term outcome data for a population that has been underrepresented in previous studies.

However, several limitations of this study should be acknowledged. First, the comparison between the patients treated before and after 2012 introduced potential era-related confounding. Improvements in systemic therapy, radiotherapy, imaging, and pathology over time may have influenced both recurrence risk and survival independently of axillary management. In addition, these developments may have contributed to improved survival outcomes for the no-ALND group treated after 2012, potentially mitigating the effect of omitted axillary surgery.^20^ Although multivariable adjustment was performed, residual confounding cannot be excluded.

Second, radiotherapy information was unavailable for the majority of the mastectomy patients in our dataset, limiting our ability to assess the potential influence of postmastectomy radiotherapy on AR and OS in this subgroup.

Third, the use of neoadjuvant systemic therapy increased during the study period, and these patients were excluded from the current analysis. This may have led to a relatively greater exclusion of higher-risk patients from the no-ALND group treated after 2012, potentially contributing to more favorable outcomes for this cohort.

In addition, 30.2% of the patients in the no-ALND group were 70 years of age or older compared with 11.6% in the ALND group. Older patients may be less likely to receive adjuvant systemic therapy and are at higher risk of death from non-cancer causes, which may influence OS independently of axillary management. Although age and adjuvant treatment were included in the multivariable analysis, residual confounding related to age and treatment selection may have remained.

Furthermore, cause of death was not available in the current dataset. The significantly lower unadjusted OS observed in the no-ALND group may therefore partly reflect the substantially older age distribution in this group and a higher rate of death from causes unrelated to breast cancer. The absence of cancer-specific survival or distant recurrence-free survival data limited the ability to determine whether differences in mortality were related to breast cancer outcomes or competing non-cancer mortality.

Finally, the median follow-up period, although substantial, was shorter for the no-ALND group (5.97 years) than for the ALND group (13.07 years). Because AR may occur late for patients with limited nodal disease, the shorter follow-up period may have underestimated the true long-term AR risk after omission of ALND.

Despite these limitations, our results provide robust, real-world evidence supporting the safety of omitting ALND for patients with minimal axillary metastases. Given the well-known morbidity associated with ALND, including lymphedema, pain, and impaired arm function, these findings support continued refinement of axillary management for appropriately selected patients.^10^

In conclusion, this large nationwide study, including a substantial subgroup of patients treated with mastectomy, with a follow-up period of 13.07 years demonstrated that omission of ALND for patients with pN1mi or pN0(i+) disease is associated with a low incidence of AR and no adverse impact on OS. These findings reinforce the safety and appropriateness of guideline-based de-escalation of axillary surgery and contribute strong real-world evidence to the evolving international consensus on contemporary axillary management.
